# β-defensins: An innate defense for bovine mastitis

**DOI:** 10.14202/vetworld.2017.990-998

**Published:** 2017-08-26

**Authors:** Ankita Gurao, Sudhir Kumar Kashyap, Ravinder Singh

**Affiliations:** 1 Department of Veterinary Microbiology and Biotechnology, College of Veterinary and Animal Sciences, Rajasthan University for Veterinary and Animal Sciences, Bikaner - 334 001, Rajasthan, India; 2 Department of Biotechnology, Sri Guru Granth Sahib World University, Fatehgarh Sahib - 140 407, Punjab, India

**Keywords:** *Bos indicus*, β-defensins, mastitis

## Abstract

Immune challenges are inevitable for livestock that are exposed to a varied range of adverse conditions ranging from environmental to pathogenic stresses. The β-defensins are antimicrobial peptides, belonging to “defensin” family and therefore acts as the first line of defense against the major infections occurring in dairy cattle including intramammary infections. The better resistance to mastitis displayed by *Bos indicus* is implicit in the fact that they have better adapted and also has more sequence variation with rare allele conserved due to lesser artificial selection pressure than that of *Bos taurus*. Among the 58 *in silico* predicted β-defensins, only a few have been studied in the aspect of intramammary infections. The data on polymorphisms occurring in various β-defensin genes is limited in *B. indicus*, indicating toward higher possibilities for exploring marker for mastitis resistance. The following review shall focus on concisely summarizing the up-to-date research on β-defensins in *B. taurus* and discuss the possible scope for research in *B. indicus*.

## Introduction

The cattle and buffaloes hold the second position among livestock population after the chickens, with largest population inventory in India [1]. This implicates toward the importance of sound health of the livestock toward sustained economy of livestock sector of the country. In contradiction, ever changing global environment has posed challenges to the livestock producers in this 21^st^ century. The major fraction of health challenges that affect livestock productivity includes exposure to the environmental stress as well as pathogenic stress.

In cattle and buffaloes, mastitis is one of the most economically devastating pathogenic conditions. The multitude of factors results in this intramammary infection nevertheless the susceptibility to this disease is determined by both the environmental factors and the genetic makeup of organism. On the other hand, the causative pathogen serves as selecting factor for evolution of the host’s resistance toward the disease condition [2]. The host’s resistance is determined by the degree of defense presented toward pathogen. The immune genes are among the candidates better known to undergo adaptive evolution due to the pathogenic exposure over centuries as shown in several species [3], especially the first line of defense comprising the innate immune genes are more deserved target have undergone massive positive or balancing selection [2,4]. It has generated genotypes that are more suited to the natural conditions. Furthermore, a number of innate immune genes have been implicated when analyzing single nucleotide polymorphisms (SNPs) in cohorts of disease resistant or susceptible food-producing animals.

The modern dairy breeds that have undergone intense selection for productivity are more prone to mastitis than the ancient breeds which mostly comprises the naturally evolved indicine cattle, viz., *Bos indicus* [3,5]. This is clear from the data depicting the Holstein and Jersey crossbreeds of India higher risk (94.54%) of mastitis than the local cattle breeds (31.25%) [6].

One of the innate immune components, β-defensins are the second most approachable defense after the first line physical barrier for preventing intramammary infection. Furthermore being an antimicrobial peptide (AMP), it could assure complete clearance of pathogens without causing inflammation in the epithelial membrane as found in the case of other AMPs, viz., cytokines. Hence, it lead to the fact that the secretory/soluble proteins’ in this cattle must display more diversity in terms of genetic polymorphism and generate more possibility for establishing marker the most prevalent disease among the dairy livestock, such as mastitis.

This review shall try to elaborate the role of the β-defensins in context of mastitis and narrate the members of the β-defensin family in cattle that have been reported till date for their association with mastitis.

### Proteins Known to be Involved in Innate Immunity in Mammary Gland

The innate immune system is a complex and dynamic concept comprising various components majorly the physical barrier, the resident cellular components, and the mobile inducible or the inflammatory responses are shown by the white blood cell (WBC) elements. The gene encoding for any of the secretory soluble protein of humoral defenses or cellular defenses display polymorphism or may undergo mutation, leading to alternate alleles, therefore, resulting in functionally different peptide. Proteins are the mediators of all kinds of humoral defenses and cellular defenses. The responses mediated by innate immune system have an advantage over humoral system since they do not require memory for the first line defense and therefore responds rapidly [7]. The pathogens are invading mammary gland, digestive tract, and reproductive tract in livestock are sensed first by surface/physical or chemical barriers of the innate immune system. After failure of the surface/physical and chemical barrier, the system reacts by initiating inflammation mediated by the resident macrophages as well as mobile neutrophils. The bovine neutrophils score highest among the cellular WBC components. Exceptionally at surface barriers some cells and glands are specialized in secreting certain kind of AMPs, e.g., histatins by salivary gland [8] α-defensins by Paneth cells, β-defensins by the pancreas and milk serum amyloid protein by mammary epithelial cells (MECs), therefore, recruiting humoral innate defense [9]. They secrete an array of AMPs to get rid of foreign invaders.

The cationic peptides are one of the massive groups of antimicrobial compounds with characteristic cationic charge [10]. These peptides are categorized majorly into four groups on the basis of structure. The first class is composed of aliphatic a-helices; the second class has the molecules with loops and single disulfide bond. The third class is amphipathic nature with extended structure, and the fourth class is comprised more than one disulfide bonds and a stable beta sheet [11]. The defensins belongs to the fourth class. The “defensins,” the term coined by Selsted *et al*. [12] belongs to fourth class of cationic AMPs otherwise known as host defense peptides (HDPs), widely distributed in plants, vertebrates, and invertebrates [13]. The defensins are cationic AMPs were first reported in rabbit lung macrophages in 1983 [14], and characterized for their primary structure [12]. Besides the antibacterial activity of defensins, antifungal [15] and antiviral properties were also described by Lehrer *et al*. [16].

### β-defensins

The β-defensins (~10 kDa) are one of the member of defensin family that are cationic and cysteine-rich AMP displaying a varied pattern of cysteine spacing and disulfide bonding between cysteine residues. This cysteine has been claimed to protect the peptide from being digested [17]. Mammalian defensins encoded generally by a bi-exonic gene. When the gene is transcribed to the corresponding mRNA, the exon-1 forms the 5’UTR region along with the signal peptide region with propeptide-encoding mRNA region, whereas the exon-2 forms the mRNA region for mature peptide and 3’-UTR region. Thus, the primary translation product has an inactive precursor (pre-propeptide) and signal sequence at the N-terminal, and at another hand the short pro-piece of C-terminal. The mature peptide is formed from the cleaving of the pro-piece. They have a signal sequence at the N-terminal and the short pro-piece at C-terminal. The mature peptide is formed after getting cleaved off from the pro-piece. Based on the bonding between the six cysteine residues and their bonding pattern, the mammalian defensins can be classified into three sub-families; α, β, and θ-defensins (Table 1). The β-defensin was isolated from bovine respiratory tract [18], the α-defensin from murine Paneth cells [13], and the θ-defensins were discovered in rhesus monkey [19].

**Table-1:** Subfamilies of mammalian defensins.

Subfamily	Structure	Cysteine bonding	Distribution	References
α	Linear	C^1^-C^6^, C^2^-C^4^, C^3^-C^5^	Mammals	Selsted *et al*., [12]
β	Linear	C^1^-C^5^, C^2^-C^4^, C^3^-C^6^	Mammals	Diamond *et al*., [18]
θ/retrocyclins	Circular	C^1^-C^6^, C^2^-C^4^, C^3^-C^5^	Rhesus*	Tran *et al*., [19]

* Reported only in Rhesus monkey among mammals

### Mechanism of β-defensins action

In the recent period, the multidrug resistance and antibiotic resistance phenomenon have become a vogue [20]. It’s quite intriguing to know the absence of such phenomenon in the case of defensins, one of the possible reasons is the production of the very little amount of the peptides at the site or sometimes the peptides are in a functionally latent form which after reaching the site of action becomes more potent [21]. The β-defensins are amphipathic cationic peptides that have been reported to function as AMPs for the Gram-negative, Gram-positive bacteria, viruses, fungi, and other unicellular parasites [22]. Conversely, the host has also coevolved with possible mechanisms for resisting the microbicidal activity of β-defensins.

Initially, the peptides interact with the membrane of the pathogen by exploiting electrostatic attraction or in some cases mediated by receptors present on the membrane [23]. After the initial interaction with the membrane, the AMPs permeabilize the target cell only when reach they reach a threshold concentration followed by peptide conformation’s transition. In several cases, this phase transition is feasible only on availing a negatively charged membrane, which again points out toward the ability to differentiate the host cell from the target [24]. For the β-defensins, the phase transition is relatively uncommon since the structure is more stable and therefore remains unchanged on interacting with target membrane [25]. Some evidence suggests self-association between 2 and more AMP peptides to form complex structures [23].

This initial binding and interaction phase are followed by permeabilization [26,27]. Some of the mechanisms that have been proposed for permeabilization include the pore model, toroidal pore model, carpet model, barrel stave model, molecular electroporation model, and the sinking raft model. The other uncommon models suggest the immunomodulatory activity of defensins which aids wound healing. This immunomodulatory function particularly makes these defensins deserving candidate for replacing conventional antibiotics to treat intramammary infections (IMIs), since it avoids inflaming the mammary epithelial tissues [28]. Apart from the membrane permeabilization, the AMPs have been reported to stimulate hydrolases, therefore degrading the cell wall [29].

### Cattle β-defensins

The first β-defensin was isolated from bovine respiratory tract and was named tracheal AMP (TAP) [18]. In 1993, 13 bovine β-defensin peptide sequences were reported by Selsted *et al*. [30], along with the *in vitro* antibacterial activities using *Staphylococcus aureus* and *Escherichia coli* as test organisms. While, in 2004, Roosen *et al*. [31] reported 18 bovine β-defensin including 6 novel genes (DEFB401, DEFB402, DEFB403, DEFB404, DEFB405, and lingual AMP [LAP] like).

**Figure-1:**
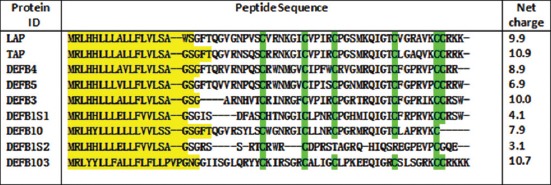
Multiple sequence alignment file for the β-defensins reviewed (the green color highlighted areas indicates the highly conserved six cysteine residues, which forms the basic definition of β-defensins and the yellow highlighted sequence are the signal peptide region for respective beta defensins).

The clustering of bovine β-defensin has been done on the basis of synteny analysis of 57 open reading frame containing the 6 cysteine containing domain (Figure-1), characteristics to the β-defensins with already published data on human, chimpanzee, mouse, rat, and dog [32,33]. The total of 58 genes within four clusters have been identified in bovine genome on chromosome number 8, 13, 23, and 27 and were designated as Cluster A, Cluster B, Cluster C, and Cluster D, respectively [34].

The Cluster A with least number of genes and Cluster D with the highest number of immunologically important genes. Whereas, the Cluster C and Cluster B have β-defensins those are expressed in the reproductive tract. The details about the clusters have been summarized in Table 2.

**Table-2:** The physical, chemical, and secondary structure predictions for the cattle β-defensins.

Name	Physiochemical parameters						Secondary structure predictions		
	
	Amino acid length	Molecular weight	Theoretical PI	Instability index	Aliphatic index	GRAVY*	α helix	β helix	Transmembrane helix
TAP	64	6953.1	10.26	18.54	103.44	0.431	33	30	25
LAP	64	7041.50	11.24	50.66	95.94	0.122	47	16	25
DEFB1S1	60	6665.98	8.98	28.16	102.33	0.447	48	18	-
DEFB1S2	54	6115.0	9.30	76.97	77.59	-0.519	46	11	-
DEFB3	60	6764.17	11.47	18.00	97.50	0.243	23	33	-
DEFB4	63	7233.74	11.46	46.73	86.51	0.278	33	25	-
DEFB5	64	7227.73	10.47	59.02	95.78	0.380	53	9	25
DEFB10	62	6954.46	10.35	39.25	114.68	0.429	50	13	-
DEFB103	67	7614.31	9.93	40.62	106.27	0.185	52	18	24

TAP=Tracheal antimicrobial peptide, LAP=Lingual antimicrobial peptide, DEFB1S1 and DEFB1S2=Splice variants of β-defensin 1 peptide, DEFB3=β-defensin 3 peptide, DEFB4=β-defensin 4 peptide, DEFB5=β-defensin 5 peptide, DEFB10=β-defensin 10 peptide, DEFB103=β-defensin 103 peptide,

* GRAVY=Grand average of hydropathicity, PI=Isoelectric point

The genes that impart resistance against intramammary infections are located on chromosome Btau 27. It implies that the Cluster D is present on Btau 27 in cattle as well as buffaloes carries a maximum number of genes of interest (Table 3). The cluster has a total of 30 genes according to the synteny analysis with human, dog, chimpanzee, and rat and mouse [32]. Only 8 of them are found to be conserved in human and dog; those are BBD105, BBD103, BBD106, BBD108, BBD104, BTSPAG11, BBD109, and BBD1. Whereas, 11 of the 30 genes are found to be specific to *Bos* genus, including LAP, TAP, EAP, DEFB4A, DEFB5, BNBD7, BNBD10, BNBD10A, BNBD11, BBD403, and BBD1 [34]. This type of phylogenetic analysis is still lacking for buffalo. Since the β-defensins are expressed by both the mammary gland and the milk somatic cells, therefore prevents the intramammary infection. Furthermore, the β-defensins are triggered by toll-like receptor (TLR) mediated nuclear factor-κB pathway, so it was assumed to be another major factor conferring resistance against mastitis in cattle and buffalo next to the TLRs [35]. Overall, the nonsynonymous SNPs subsequently leading to the modifications in amino acids’ sequence potentially improve the antibiotic activity of the peptide [36]. It may be possible that the better resistance in indicine cattle is due to the variations occurring in the coding region of these genes.

**Table-3:** Clusters of β-defensin in cattle (adapted from Meade *et al.*, [24]).

Cluster	Chromosome no.	Genes number(s)	Expressed in tissue/organ(s)	Function(s)
A	Btau 8	4	Unknown	Unknown
B	Btau 13	19	Reproductive tract	*In vitro* shown to have antimicrobial activity. *In vivo* not reported
C	Btau 23	5	Unknown	Unknown
D	Btau 27	30	Neutrophils, macrophages and epithelium tissues	Inducible in response to LPS and pathogens such as *E. coli* and *Staphylococcus aureus.* Association detected between SNPs in these gene(s) with SCC, milk yield, fat, protein lactose, and coat color in Holstein cattle

SNP=Single nucleotide polymorphisms, LPS=Lipopolysaccharide, SCC=Somatic cell count, *E. coli=Escherichia coli, S. aureus=Staphylococcus aureus*

**Figure-2:**
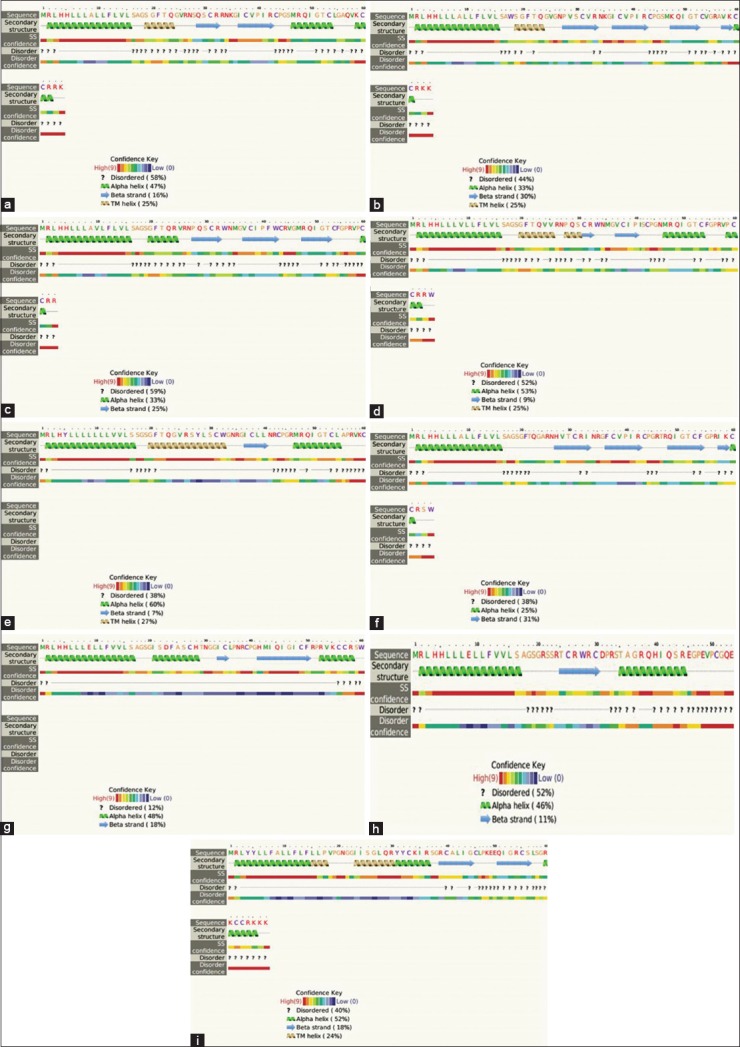
Secondary structures for the β-defensins, (a) lingual antimicrobial peptide (LAP), (b) tracheal antimicrobial peptide (TAP), (c) β-defensin 4, (d) β-defensin 5, (e) β-defensin 10, (f) β-defensin 3, (g) β-defensin 1 V1 (Isoform 1), (h) β-defensin 1V2 (Isoform 2), (i) β-defensin 103 (the confidence key and figure indices provided in the inset of respective figures).

The present review shall focus on the β-defensin whose expression is localized to the mammary gland and milk somatic cells. They could be elected as a marker for selection of cattle with resistance to mastitis. The list of β-defensin (Table 4) genes along with their significance described in the following section. Whereas, the general physical and chemical properties along with the secondary structure predictions of the reviewed defensins are listed in Table 2. The individual secondary structure has been illustrated for LAP, TAP, DEFB1 SV1, DEFB1 SV2, DEFB 5, DEFB 10, and DEFB 103 (Figure-2) [37].

**Table-4:** The list of gene in mammary gland and their clinical relevance (adapted from Roosen *et al*., [31]).

Gene	Expressed tissue/cell	Lactating status and clinical finding (or) treatment^^#^
LAP	MGT* MSC^@^	J-H, L-H, L-I, NL-H* HCD diet^
TAP	MGT* MEC^#^	L-H*, *S. aureus in vitro* challenge^#^
DEFB3	MGT* MSC^@^	L-H*, NL-I*
DEFB4	MGT^θ^* MSC^@^	L-I*, *S. aureus in vivo* challenge^θ^
DEFB5	MGT^θ^* MSC^@^	L-I*, *S. aureus in vivo* challenge^θ^
DEFB1	MGT^θ^	*S. aureus in vivo* challenge^θ^
DEFB103	Other than mammary gland**	NA

* Roosen *et al*., [31],

# Lopez-Meza *et al*., [44],

@ Bagnicka *et al*., [53],

^ Jin et al., [41],

θ Cormac *et al*., [50],

** Mirabzadeh-Ardakani *et al*., [59]. TAP=Tracheal antimicrobial peptide, LAP=Lingual antimicrobial peptide, DEFB3=β-defensin 3, DEFB4=β-defensin 4, DEFB5=β-defensin 5, DEFB1=β-defensin 1, DEFB103=β-defensin 103, MGT=Mammary gland tissue, MSC=Milk somatic cells, J=Juvenile, L=Lactating, NL=Non-lactating, H=Healthy, I=Infected, *S. aureus=Staphylococcus aureus*

### LAP

It was the first isolated from inflamed squamous tongue epithelia [38]. Apart from the tongue epithelium cells, it was found to be expressed in infected intestinal, respiratory tissue and mammary epithelial tissue [39,40]. A LAP like peptide was characterized, having high sequence similarity with the LAP [31]. Although the LAPs expression was found to be very little in squamous tongue epithelial and respiratory tissue, it is very high in the intestinal tissues. In the mammary gland constitutive expression of LAP has been reported in juvenile, lactating (both healthy and infected) and non-lactating cattle [31]. Whereas, another study has shown the LAPs expression only in infected cattle [40]. In the study conducted by Swanson *et al.*, [40], the mammary gland was infected by infusing the wild type strain of *Streptococcus uberis* and the alveolar, external and peripheral tissues of mammary gland were analyzed, whereas Roosen *et al*. [31] have studied the parenchymal and external tissue of cattle diagnosed for clinical mastitis. The type of diet also influences the level of LAP expression in the mammary gland. The high concentrate diet has been reported to enhance the expression of LAP [41]. The direct relationship between one of the β-defensin peptide, LAP and somatic cell count (SCC) have been accounted, where they have shown higher concentration of LAP in the milk of cattle infected with *S. aureus*, *Streptococcus bovis*, *Streptococcus dysgalactiae*, and *E. coli* than that of uninfected one [42]. Apart from that, on the basis of the expression studies, the LAP has been mapped out as genetic marker for mastitis trait associated marker in cattle [36,43].

### TAP

It was the first bovine β-defensin to be isolated from mammalian tracheal mucosa [18]. The TAP has been reported to be expressed in mammary cells of both infected and healthy (Table 4) [31]. The expression of TAP has also been shown in the bovine MEC (bMEC) *in vitro* infected with *S. aureus* [44]. In contrast to the above discussion, during an experimentally induced *S. aureus* infection in cattle, the expression of LAP, as well as TAP, was found to be very low or negligible [45]. The expression of the TAP, as well as the LAP, is regulated by Oct-1 transcription factor [46].

A nonsynonymous SNP in Gir and Murrah buffalo three nonsynonymous SNPs have been reported by Patel *et al*. [47]. The huge increment in the number of nonsynonymous SNPs in buffalo could significantly alter the primary structure of the β-defensin in buffalo as compared to cattle. Nonsynonymous SNPs in exon 2 have been reported by Ryan *et al*. [48], which modulates the bactericidal activity. Interestingly, the haplotype analysis has shown a higher number of haplotypes in buffalo for the TAP gene in comparison to the cow breed(s) and also the linkage disequilibrium value for buffalo has been found higher than *Bos* species. Therefore, indicating toward the functional relevance of the SNPs in buffalo.

### DEFB1

The two variants of β-defensin 1 are DEFB1V1 and DEFB1V2. Alternatively, the gene is known as BNBD1, is one of the inducibly expressed β defense during intramammary gland infection [31]. The majority of SNPs reported in indicine cattle lies in 5’UTR and a noteworthy start gain, mutation reported, whereas in Murrah buffalo 2 such start gain, mutation have been reported in the same study [47].

### DEFB4

The DEFB4 also known as BNBD4 was first reported in 1993 [30]. Their expression was initially found constitutive and higher in alveolar tissues in bovine. Whereas in small intestine its expression was too lower [49]. Another study concluded the higher level of expression of DEFB4 in alveolar and cistern tissue of mammary gland challenged with *Staphylococci aureus* [45,50]. Yet, another study has also indicated the increased expression of DEFB4 or BNBD4 in mammary gland infected with coagulase-positive *Staphylococci* [48] than those of coagulase-negative *Staphylococci* [51]. This difference may be due to the different mode of permeabilizing the bacterial membrane in Gram-positive and Gram-negative bacteria [27].

A great extent of SNP has been reported in the intronic region of DEFB4 in *Bos taurus* [52]. The 2 out of the 10 SNPs reported were found to be associated with milk composition traits and SCC. This establishes DEFB4 as a marker for mastitis resistance [53].

### DEFB3

The BNBD3 or DEFB3 was found to be expressed in various tissue types. A very high level of such expression has been observed in bone marrow [54]. An interesting finding has indicated the elevated level of expression in BNBD3 alias (DEFB3) in the bovine monocyte culture treated with lipopolysaccharide in a dose-dependent manner [55].

### DEFB5

The DEFB5 or BNBD5 has been reported which resembles the DEFB4 [30]. Its expression was initially shown to be higher in the macrophages located on the surface of bovine pulmonary alveoli and also the higher level of expression of β-defensin gene (i.e., BNBD5) in MEC has been demonstrated during intramammary infection [56,57]. An another study has also concluded with even higher level of expression of DEFB5 than the DEFB4 in alveolar, ductal and cistern tissue of mammary gland challenged with *S. aureus* [45,50].

The increased expression level of both DEFB5 and DEFB4 has been reported in mammary gland infected with coagulase-positive *Staphylococci* than those of coagulase-negative *Staphylococci* [51], however in the case of DEFB5 only the cow in later lactation stage showed this higher level of expression in contrast to DEFB4 which showed higher expression even in early lactation stage. While no such polymorphism in DEFB5 has been reported significant in indicine cattle and buffalo [47].

### DEFB10

The expression of DEFB10 has been observed higher in early lactation stages than in the later stages on infection by coagulase-positive *Staphylococci*. Whereas for DEFB1, BNBD4, LAP, and BNBD5 expression were found to be higher in later stages on being inoculated by coagulase-positive *Staphylococci* [51]. One of the studies conducted has shown an elevated level of DEFB10 expression in the presence of sodium octanoate when bMECs were challenged with *S. aureus* subspecies *aureus* (ATCC 27543) [57]. However, in the case of *Mycoplasma bovis* infection, the DEFB10 expression was reported to be down-regulated [58].

### DEFB103

DEFB300 or DEFB103A or DEFB103B is a newly found β-defensin on the BTAU 27. It shares very little homology with the other members of Cluster D β-defensin within the species and also when compared with other species [59]. Its expression has been very little studied in cattle. The highest level of DEFB103 expression in buccal epithelium among all other tissues (nictitating membrane, rumen, shoulder skin, and bladder) analyzed [59]. No, any report presents its expression in the mammary gland.

Five new SNPs have been reported in 5’UTR region of DEFB103 [60]. Another couple of SNPs were reported later [61]. Moreover, similarly, the authors have described no significant association of the four haplotypes on DEFB103 polymorphism with resistance or susceptibility to mastitis caused by *S. aureus* [59].

## Future Scopes

### Alternative to antibiotics

Worldwide the dairy industry spends a huge amount on combating against the intramammary infections. The antibiotics have been the major therapeutic drugs used for treatment. On the other hand, the efficiency of antibiotics has declined over the period due to repeated and improper use. Hence, the best strategy to treat mastitis should involve exploiting the innate potential of the host to fight against infections, especially the AMP like β-defensins. It is noteworthy that no such resistance has been developed by microbes against these AMPs since the target for these AMPs are the microbe’s integral structure and also it influences the host system by immunomodulation [33,62,63]. The immunomodulatory role of these defensins aids not only augments the direct antimicrobial mechanism but also repair the epithelial surface where the AMPs were expressed by Lai and Gallo [64]. Therefore, recently an approach was devised to prepare synthetic peptides called innate defense regulator peptides (IDRs) [65], that resembles some functionally active sequences of the AMPs. This may be considered as a potential tool for mastitis treatment if the reported functional defensins during mastitis are considered. In an another approach site-directed mutagenesis was used to bring targeted modification in amino acid sequence of porcine β-defensin 2, subsequently leading to a more effective AMP. This could serve as a tool to improve the antimicrobial activity of some bovine β-defensins, such as TAP and LAP.

### Adjuvants for improved vaccine design

The vaccine for mastitis has been a point of discussion. The β-defensins are one of the desired candidates for vaccine adjuvants, since these peptides are known to stimulate the Th1 response and also the Th2 responses and impart immunoadjuvant effect [66]. Several defensins have been earlier reported to function as adjuvant. Earlier a vaccine for bovine herpesvirus 1 made from bovine neutrophil β-defensin 3 conjugated with glycoprotein D as an adjuvant has also been reported by Mackenzie-Dyck *et al*. [67].

### β-defensin expression and the dietary influence

The immune system of an individual is widely influenced by nutrition. In the case of β-defensins, several studies have shown modulation of HDPs expression under different dietary conditions. In humans, the dietary histone deacetylase inhibitor sulforaphane and sodium butyrate (short chain fatty acid) have been reported to upregulate the human β defesin-2 expression in human caco-2, HT-29, and SW480. Later the sodium butyrate to be linked with higher expression of HDPs in piglets infected with *E. coli* and also reported lowering of *E. coli* load [63]. Therefore, the diet could itself contribute toward better resistance for mastitis and lower the medical costs.

## Conclusion

Although the milk production has gained a hike after white revolution, but introducing the exotic breeds (*B. taurus*) has loaded the Indian dairy sector with the curse of mastitis and subsequent economic burden of the treatment. The mastitis, like any other pathogenic condition, is indispensably linked to lower resistance of host to the pathogens. The β-defensins offers three tier solutions for this menage. The level one of remedy involves screening the more ancient breeds or in other words, the breeds that have been lesser manipulated for milk yield, e.g., *B. indicus* for SNPs in coding region of β-defensin genes on 27^th^ BTAU. The aforesaid screening could serve as tool for selecting more resistant animals since the β-defensin are the best known genetically encoded antimicrobials. The second tier of solution involves using the β-defensins peptides as adjuvant to deliver vaccine for mastitis, although up to date no vaccine for mastitis has been developed which uses these peptides as an adjuvant. The third tier solution derived from the β-defensin enables synthesizing the IDRs by getting clue from naturally occurring potent acting β-defensins from either species of *Bos* genus. Otherwise manipulated version of these peptides may also be generated to design IDRs. In a concluding statement, it is not false to say that synthesized antimicrobials do have better competitors already existing in nature since millions of years; therefore, the research could be an exploratory task to solve multifactorial problems like mastitis.

## Authors’ Contributions

All authors contributed extensively in drafting and revision of the manuscript. AG and SKK reviewed the existing literature and critically analyzed the data. SKK has structured the whole manuscript, and RS performed bioinformatics analysis. All authors read and approved the final manuscript.
